# A survey of registered pharmacological clinical trials on rare neurological diseases in children in 2010–2020

**DOI:** 10.3389/fped.2022.963601

**Published:** 2022-11-04

**Authors:** Xuting Chang, Jie Zhang, Yuwu Jiang, Meixia Shang, Ye Wu

**Affiliations:** ^1^ Department of Pediatrics, Peking University First Hospital, Beijing, China; ^2^ Department of Medical Statistics, Peking University First Hospital, Beijing, China

**Keywords:** rare neurological diseases, children, clinical trials, study design, methodological analysis

## Abstract

**Objective:**

To clarify the current state of methodology of clinical trials for rare neurological diseases in children, and to provide a basis for the further optimization of the trial design.

**Methods:**

Data of clinical trials for the rare neurological diseases with childhood onset (searched through https://rarediseases.info.nih.gov/diseases and www.Orpha.net) registered on the Clinicaltrils.gov from January 2010 to June 2020 was collected. Analysis on the methodology of the clinical trials were performed, focusing on initiator of the studies, multi or single research center, study design, sample size, and the endpoint using in the trial.

**Results:**

A total of 162 clinical trials were included, covering only 7.3% (61/835) of rare neurological diseases in children. 101 (62.3%) were initiated by pharmaceutical companies, and 61 (37.7%) by investigators. Most (95.4%) of global multicenter studies were initiated by pharmaceutical companies, whereas most (70.0%) of single-center studies were initiated by investigators (*χ*^2 ^= 61.635, *P* < 0.001). Of the 162 trials, 74 (45.7%) were open-label single-arm trials, 68 (42.0%) were randomized double-blind parallel controlled trials (RCT), 12 (7.4%) were randomized crossover trials. Most of RCTs (73.5%) and 54.1% of open-label single-arm trials were initiated by pharmaceutical companies. The proportion of RCTs in clinical trials for diseases with a prevalence of ≥1/10,000 (62.5%) was higher than that in diseases with prevalence ≤1/1,000,000 (12.0%) or 1/1,000,000~1/10,000 (43.1%) (*χ*^2 ^= 14.790, *P* = 0.001). The median expected sample size of the studies was 34 (4–500). 132 (132/162, 81.5%) studies enrolled fewer than 100 cases. Diseases with a prevalence of ≥1/10,000 had significantly larger sample sizes than other prevalence classes (*P* < 0.001, *P* = 0.003).

**Conclusions:**

There were few clinical trials targeting on treatment of rare neurological diseases in children. Trials on rare diseases used fewer participants, and high-quality randomized controlled trials were less common. It is necessary to conduct global multicenter recruitment and choose optimal study designs to improve the level of evidence in clinical trials on rare diseases.

## Introduction

A rare disease is defined as a disease affecting a limited number of people. Definitions of rare diseases vary in different countries. In the United States, a rare disease is defined as one that affects fewer than 200,000 individuals nationwide. In the European Union, a rare disease is one that has a prevalence of no more than 5 in 10,000 individuals or affects fewer than 250,000 individuals. In Japan, a rare disease is one that affects fewer than 50,000 patients (1). In China, a rare disease is one that affects fewer than 140,000 individuals or has a prevalence of no more than 1 in 10,000 individuals or has an incidence of less than1 in 10,000 newborns. Approximately 7,000 rare diseases are recognized at present, and approximately 6%–8% of individuals suffer from rare diseases worldwide. More than 50% of cases with rare diseases begin in childhood (2). Currently, only 5% of rare-disease cases can be effectively treated. Although many countries have announced relevant policies encouraging the research and marketing of “orphan drugs”, there are still few prospective trials of treatments for rare diseases. Ryuichi Sakate et al. (3) conducted a study in which they searched the ClinicalTrials.gov for all clinical drug- intervention trials targeting rare diseases in 1999–2017, only 1,535 diseases were studied, 70% of which were studied in fewer than 10 trials, and most of the clinical trials were on rare cancers.

There are many challenges in the conduct of clinical drug- intervention trials for rare diseases. The number of patients is limited, and the patient populations are frequently geographically dispersed, making it difficult to recruit subjects for clinical trials. Furthermore, the lack of information regarding the natural history of some of these diseases and appropriate endpoints make it difficult to conduct high-quality studies such as randomized double-blind parallel controlled trials (4); thus, researchers must consider how to design high-quality studies with limited numbers of patients. In addition to the above problems, clinical drug intervention trials in children are further complicated by the incomplete state of liver and kidney development in children, which makes their pharmacokinetics quite different from that of adults, and by the ethical challenges imposed by children's disadvantaged status. However, more than half of patients with rare disease begin in childhood; therefore, it is important to carry out clinical trials for drug development in children.

In this study, we analyzed the methodology of the clinical trials registered in ClinicalTrials.gov for rare neurological diseases in children in 2010–2020, to clarify the current state of clinical trials for rare diseases, and to provide a basis for the further optimization of the trial design.

## Material and methods

### Information sources and eligibility criteria

**Figure 1:**
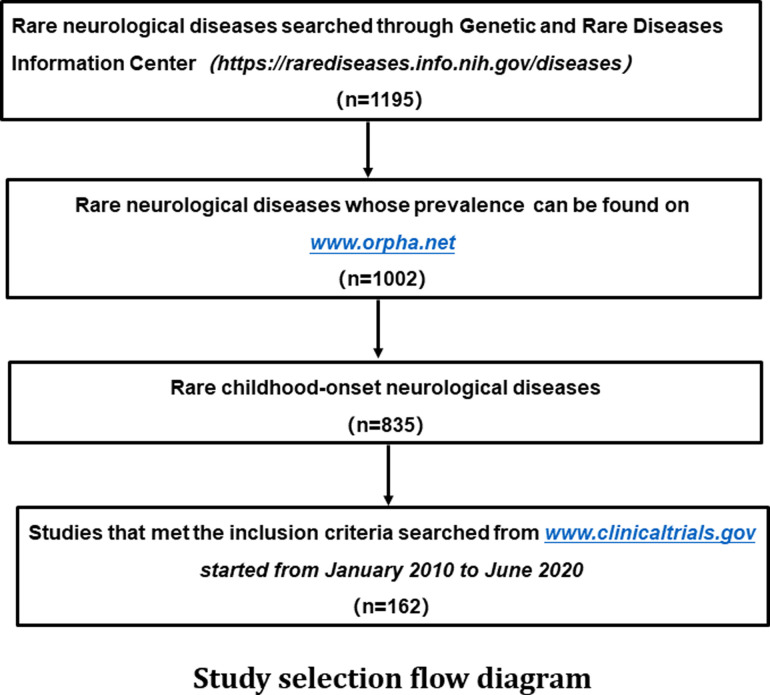
**Study selection flow diagram** A total of 1,195 rare neurological diseases were identified through the *genetic and rare diseases information center* (https://rarediseases.info.nih.gov/diseases), of which 1,002 diseases could be found on www.Orpha.net. A total of 835 diseases affected either children only or both children and adults. Further search in ClinicalTrials.gov found a total of 162 clinical trials on above diseases were registered from January 2010 to June 2020.

We followed the PRISMA Statement guidelines for conducting the study when the items are applicable (5). We first searched for rare neurological diseases in the resources of the *Genetic and Rare Diseases Information Center* (https://rarediseases.info.nih.gov/diseases). Next, we searched www.Orpha.net for the prevalence and age of onset of these diseases to identify rare neurological diseases with childhood-onset. Finally, we screened on the ClinicalTrials.gov for child-specific drug clinical trials for the above rare childhood-onset neurological diseases started from January 1, 2010 to June 30, 2020, as the clinical trials included in this study (Figure 1).

### Data collection

Relevant information from each clinical trials was extracted, including sponsor of the clinical trial, the disease, the pathogenetic mechanism of the disease, whether the clinical trial was internationally multi-center, study design, the sample size (relation to the prevalence of the rare disease), the medications in the clinical trials, the endpoint of the study and the status of the trial. Two investigators (Chang Xuting and Zhang Jie) independently searched and selected the studies and did data collection.

### Statistical analysis

Categorical variables are presented as frequencies and percentages. For continuous variables, the Shapiro-Wilk test was applied to test whether the measurement data followed the normal distribution. If the data did not follow the normal distribution, they were presented as the median (minimum value - maximum value); otherwise, they were presented as the mean ± standard deviation. The relationship between the study sponsor (including pharmaceutical companies and investigators) and the research center (including single-center studies, global multicenter studies, and national multicenter studies) was analyzed through the chi-square test. The relationship between the study design and drug class (including marketed and new medications) or study sponsor were analyzed through Fisher's exact test. When analyzing the relationship between prevalence and the study design method, we divided the prevalence into three group: ≤1/1,000,000, 1/1,000,000~1/10,000, and ≥1/10,000. The study designs were divided into two groups, namely, randomized double-blind parallel controlled trials (RCTs) and non-RCTs, and the chi-square test was then used for further analysis. When analyzing the relationship between prevalence of the disease and the sample size of the trial, we divided prevalence rates into three groups, namely, ≤1/1,000,000, 1/1,000,000~1/10,000, and ≥1/10,000, and the Kruskal–Wallis *H* test was then used for further analysis. All statistical analyses were conducted on SPSS (version 26.0). *P* < 0.05 was considered statistically significant.

## Results

### Only one hundred and sixty-two clinical trials were included

**Figure 2:**
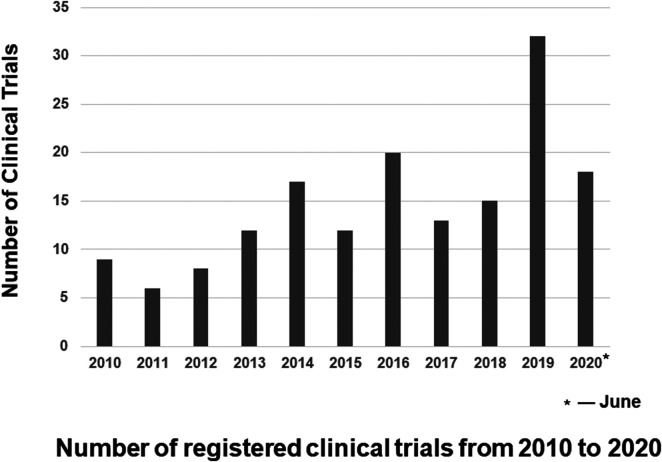
**Number of registered clinical trials from 2010 to 2020** the number of clinical trials generally increased from 2010 to 2019, and it peaked in 2019 with 32 clinical trials. Because we include clinical trials registered from 2010 and June 2020, the number of clinical trials registered in 2020 is the number of clinical trials registered before June 2020.

A total of 1,195 rare neurological diseases were identified through the *Genetic and Rare Diseases Information Center*, of which 1,002 diseases could be found on www.Orpha.net*.* A total of 835 diseases (835/1002, 83.3%) affected either children only or both children and adults. Further search in ClinicalTrials.gov found a total of 162 clinical trials on above diseases registered from January 2010 to June 2020, which were included in our analysis of methodology (Figure 1, Supplementary material Table S1). The number of clinical trials generally increased from 2010 to 2019, and it peaked in 2019 with 32 clinical trials (Figure 2). The 162 clinical trials involved a total of 61 diseases, which means that only 7.3% (61/835) of rare neurological diseases in children have drug trials.

The classification of the diseases included neuromuscular disease, hereditary metabolic disease, congenital malformations, hereditary ataxia, neurocutaneous syndrome, cranial neuropathy, special infection of rare pathogens, tumor, epileptic encephalopathies, immunodeficiency with central nervous system (CNS) involvement, neurodegenerative disease, hereditary leukoencephalopathies, CNS demyelinating disease, brain developmental disorders and other diseases. Clinical trials on metabolic diseases (23.5%, 38/162) and neuromuscular diseases (33/162, 20.4%) were the most common. And for specific diseases, clinical trials on Duchenne muscular dystrophy (16/162, 9.9%), Rett syndrome (11/162, 6.8%), Spinal muscular atrophy (10/162, 6.2%) and Fragile × syndrome (9/162, 5.6%) were the most common.

### Initiator of the study and whether it is a multicenter study

Of the 162 clinical trials, 101 (62.3%) were initiated by pharmaceutical companies, and 61 (37.7%) were initiated by investigators. A total of 43.2% of clinical trials (70/162) were single-center studies, 40.1% (65/162) were global multicenter studies, and 27 (27/162, 16.7%) were national multicenter studies. Most (95.4%,62/65) global multicenter studies were initiated by pharmaceutical companies, whereas most (70.0%, 49/70) single-center studies were initiated by investigators (*χ*^2 ^= 61.635, *P* < 0.001) (Table 1).

**Table 1:** Information of 162 clinical trials.

*N* = 162		Number of trials (%)
Initiator of the study	Pharmaceutical companies	101 (62.3%)
	Investigators	61 (37.7%)
Study center	Single-center studies	70 (43.2%)
	National multicenter studies	65 (40.1%)
	Global multicenter studies	27 (16.7%)
Prevalence of the disease	≤1/1,000,000	25 (15.4%)
	1/1,000,000~1/10000	102 (63.0%)
	≥1/10000	32 (19.8%)
	Undefined	3 (1.9%)
Study design	Open-label single-arm trial	74 (45.7%)
	Randomized double-blind parallel controlled trial	68 (42.0%)
	Randomized crossover trial	12 (7.4%)
	Randomized placebo-phase design	4 (2.5%)
	Single-arm single-blind trial	2 (1.2%)
	Ranking and selection trial	2 (1.2%)
Primary endpoints	Symptomatic improvement	29 (17.9%)
	Scale-based scores	62 (38.3%)
	Disease-specific test	19 (11.7%)
	Imaging examination	14 (8.6%)
	Safety	33 (20.4%)
	Pharmacokinetic indicator	3 (1.9%)
	Survival rate	1 (0.6%)
	Effective rate	1 (0.6%)

### Medications in the clinical trials

The 162 studies involved a total of 138 medications, including small-molecule drugs (83/138,60.1%), antisense oligonucleotides (6/138,4.3%), gene and cell therapies (12/138,8.7%), antibody therapies (11/138,8.0%), enzymes replacement therapies (9/138,6.5%) and other therapies (17/138,12.3%). Fifty-seven (35.02%) studies concerned extended indications for marketed medications, and 105 (105/162, 64.8%) were studies on new medications. Overall, 52.4% (55/105) of studies of new drugs were global multicenter studies, whereas only 15.8% (9/57) of the studies on the extended indications of marketed drugs were global multicenter studies (*χ*^2 ^= 24.552, *P* < 0.001). A total of 83.8% (88/105) of studies of new drugs were initiated by pharmaceutical companies, while 75.4% (43/57) of the studies on extended indications for marketed drugs were initiated by investigators (*χ*^2 ^= 55.613, *P* < 0.001).

### Study design

Of the 162 trials, 74 (45.7%) were open-label single-arm trials, 68 (42.0%) were randomized double-blind parallel controlled trials, 12 (7.4%) were randomized crossover trials, 4 (2.5%) used a randomized placebo-phase design (RPPD), 2 (1.2%) were single-arm single-blind trials, and 2 (1.2%) were ranking and selection trials. As for the control conditions of RCTs, 92.6% (63/68) of trials used placebo, 2 trials used active comparators (another medication), 1 trial used active control and placebo control at the same time, and the other two trials were dose-response control.

Most of RCTs (73.5%, 50/68) were initiated by pharmaceutical companies. Of the 74 open-label single-arm trials, 54.1% (40/74) were initiated by pharmaceutical companies, and 45.9% (34/74) were initiated by investigators. In addition, the proportion of trials on new drugs was slightly higher among randomized double-blind parallel controlled (69.1%) trials than among open-label single-arm trials (59.5%). The proportion of RCTs was 62.5% in clinical trials for diseases with a prevalence of ≥1/10,000, which was higher than that in diseases with prevalence ≤ 1/1,000,000 (12.0%) or 1/1,000,000~1/10,000 (43.1%) (*χ*^2 ^= 14.790, *P* = 0.001).

### The relationship between prevalence of the disease and sample size

The median sample size of the studies was 34 (4–500). One hundred (100/162, 61.7%) studies enrolled fewer than 50 cases, 132 (132/162, 81.5%) studies enrolled fewer than 100 cases, and 151 (151/162, 93.2%) studies enrolled fewer than 200 cases. Only 11 (11/162, 6.8%) studies enrolled more than 200 cases; 9 of these trials were global multicenter studies of new drugs and were initiated by pharmaceutical companies. The lowest prevalence of diseases involved in the 162 studies was less than 1/1,000,000, and the highest was 1–5/10,000. There were 25 (25/162, 15.4%) trials studying diseases with ≤1/1,000,000 prevalence, 102 (102/162, 63.0%) studying diseases with 1/1,000,000~1/10,000 prevalence, and 32 (32/162, 19.8%) studying diseases with ≥1/10000 prevalence. The median expected sample sizes were 23 (4–62), 33 (5–340), and 66 (11–500) for prevalence ranges of ≤1/1,000,000, 1/1,000,000~1/10,000 and ≥1/10,000, respectively. Diseases with a prevalence of ≥1/10,000 had significantly larger sample sizes than other prevalence classes (*P* < 0.001, *P* = 0.003).

### Primary endpoints

The proportion of patients showing symptomatic improvement was specified as the primary endpoint in 29 (29/162, 17.9%) trials. Scale-based scores, disease-specific tests, and imaging examinations were specified in 62 (62/162, 38.3%), 19 (19/162, 11.7%) and 14 (14/162, 8.6%) trials, respectively. Safety and pharmacokinetic indicators were also specified as primary endpoints in other clinical trials.

## Discussion

Although the number is increasing year by year, there were only a small number of rare neurological diseases in children (7.3%, 61/835) having registered clinical drug trials in 2010–2020. Among the 162 clinical trials included in our study, Duchenne muscular dystrophy, Rett Syndrome, spinal muscular atrophy and fragile × syndrome were the most common diseases. To clarify the current state of methodology of clinical trials for rare diseases, we conducted an analysis on these clinical trials.

In terms of initiator of the clinical trials, most (101/162, 62.3%) were initiated by pharmaceutical companies, which consistent with the findings of a previous study on rare diseases (6). Among them, in the international multi-center studies (62/65, 95.4%), new drug studies (88/105, 83.8%), or RCTs (50/68, 73.5%), studies initiated by pharmaceutical companies had the highest proportion. These findings suggested that the participation of pharmaceutical companies was essential for high-quality drug interventional study. The majority (43/61, 70.5%) of studies on repurposing use of marketed drugs were initiated by investigators, suggesting that the clinical experience and practice of investigators are also crucial for drug intervention on rare diseases, which can provide evidence for pharmaceutical companies to conduct high-quality randomized controlled trials on repurposing medications.

The small number of patients is one of the major difficulties in carrying out clinical trials on rare diseases. The median sample size of the 162 trials was only 34. Overall, 61.7% of the trials enrolled fewer than 50 cases, and 81.5% of the trials enrolled fewer than 100 cases, which was similar to the findings of Stuart A Bell and Catrin Tudur Smith on rare diseases (7), with 61.7% of the trials fewer than 50 cases, and 83.3% of the trials fewer than 100 cases in their study. However, the trials on non-rare-diseases registered on ClinicalTrials.gov enrolled more patients, and only approximately 38.2% of the trials enrolled fewer than 50 patients (7). Sample size was related to disease prevalence, with significantly larger samples for diseases with ≥1/10,000 prevalence than for other prevalence classes, corroborating the findings of Hee SW et al. (8). In short, the low prevalence of rare diseases and the scattered geographical distribution of the patients make it difficult to enroll sufficient subjects. We can address these challenges by increasing sample size through global and multicenter recruitment, establishing a recruitment platform for drug interventional studies on rare diseases, and strengthening cooperation among research institutions and hospitals. In addition, study designs must be improved to efficiently use the limited sample sizes available.

Of the 162 trials, 45.7% were open-label single-arm trials, and 42.0% were randomized controlled trials. The proportion of nonrandomized controlled studies was higher for diseases with lower prevalence. However, only 29.6% of trials for common diseases were single-arm trials in a previous study (7). The proportion of high-quality clinical trials is lower for rare diseases than for common diseases, probably because rare diseases are characterized by a small, often geographically dispersed set of eligible study participants and frequently limited understanding of the natural history of the disease; thus, it is important to choose an appropriate study design to maximize the use of the limited number of patients. Alternative clinical trial designs for studying treatments of rare diseases include randomized controlled trials, crossover and N-of-1 trials, randomized placebo-phase designs, randomized withdrawal designs, group sequential designs, factorial designs and response-adaptive randomization designs (9). In crossover trials, each participant receives all compared treatments in a randomly selected order. Since the effects of two or more treatments can be observed in the same individual, it can effectively control irrelevant variables and thus reduces the sample size needed. However, this design is appropriate only for chronic and stable conditions (10). Crossover designs were adopted in 12 of 162 studies, which were related to Rett syndrome, spinal muscular atrophy, alternating hemiplegia, and fragile × syndrome; all of these diseases progress slowly. N-of-1 trials are clinical trials based on multiple iterations of a crossover design in a single subject. The subjects receive two or more different treatments in randomized order over several crossover periods. The advantages and disadvantages of N-of-1 trials are similar to those of crossover trials. Both are appropriate only for chronic and stable conditions, and the treatment effect should be rapid in onset and rapidly reversible (11). N-of-1 trials have provided a basis for the treatment of many diseases, such as asthma, rheumatoid arthritis and cystic fibrosis; Kim J et al. (12) conducted a study on the efficacy of milasen on a child with Batten disease using an N-of-1 design method. In a randomized placebo-phase design, subjects are randomly divided into an experimental group and a control group in the early stage; later, all subjects receive therapy (13). Time to response is used as the primary outcome in a randomized placebo-phase design. Group sequential trials are similar to randomized controlled trials but allow interim analysis (8). The subjects are randomly divided into an experimental group and a control group, and then an interim analysis is performed. If the termination criterion is not met, a second group of subjects is recruited and randomized, and the study continues in this manner until the termination criterion is reached. Group sequential trials require a smaller sample size than RCTs, and the treatment effect should have a rapid onset. For some rare diseases with strong heterogeneity, a randomized withdrawal design can also be adopted (14). For research that needs to compare the efficacy of multiple treatments at the same time, a factorial design can be used (15). Of the 162 trials included in our study, there were few studies using the above-mentioned study design methods, suggesting that the participation of experts on methodology is essential in the design of clinical research on rare diseases.

As for the endpoints, the FDA defines a clinical endpoint as one that indicates how a patient “feels, functions, or survives”. “Feels” refers to the patient's clinical symptoms, such as the improvement of seizures in patients with epilepsy. “Functions” refers to the measurement of ability; for example, walking ability can be measured by a 6 min walk test. “Survives” refers to overall survival. However, if the disease progresses slowly, if the improvement of ability is difficult to quantitatively evaluate, or if the early effectiveness of the treatment needs to be examined, biomarkers can also be used as primary endpoints (16). Most of the 162 studies used symptom improvement, scale-based scores, imaging examinations, and pharmacokinetic indicators as the primary endpoints. However, it is difficult to select appropriate endpoints for clinical trials of some rare diseases because their natural history and pathogenesis are poorly understood. Therefore, it is necessary to accelerate research on the natural history and pathogenesis of rare diseases. Marielle G et al. (17) suggested that patient and caregiver video interviews can also be used to complement the data captured by traditional endpoints in clinical trials for rare diseases.

The present study had certain limitations: (1) The rare diseases included in this study was limited to those that were listed by the Genetic and Rare Diseases Information Center and the Orpha.net website, some rare neurological diseases in children may not have been included on those sites and may be missed; (2) All information on the included clinical trials was obtained from ClinicalTrials.gov, the protocols listed in the website may differ from the actually used in clinical trials; (3) The search was limited to 2010–2020, more recent studies that were registered after 2020 were missed; (4) The results were based on the clinicaltrials.gov, other national and international registries and published literature were not included, so there are concerns about generelasiability in our study.

## Conclusions

There are few clinical trials targeting on treatment of rare neurological diseases in children. Trials on rare diseases used fewer participants than those that focus on non-rare diseases, as the number of available participants is related to the prevalence of the disease. In addition, studies on rare diseases tend to use relatively simple research designs, and high-quality randomized controlled trials are less common. It is necessary to conduct global multicenter recruitment and choose optimal study designs to improve the level of evidence in rare disease trials. In summary, any successful effort to accelerate the development and application of “orphan drugs” will require the participation of doctors, pharmaceutical companies, government agencies, clinical research methodologists, biologists, patients and their families.

## Data Availability

The original contributions presented in the study are included in the article/**Supplementary Material**, further inquiries can be directed to the corresponding author/s.
