# Supplementary material for: A survey of registered pharmacological clinical trials on rare neurological diseases in children in 2010–2020
Source: Front Pediatr. 2022 Nov 4;10:963601. doi: 10.3389/fped.2022.963601 (PMC9672510; doi:10.3389/fped.2022.963601)
Supplement: Supplementary file 1 [file Datasheet1.docx]

| **ID** | **Disease** | **Medication** | **Pathogenetic mechanism** | **Whether the drug targets the pathogenic mechanism** | **Study design** | **Prevalence** | **Sample size** | **Primary endpoint** | **Original primary endpoint** | **Initiator** | **Status of the trial（Date）** | **PMID/NCT number** |
| --- | --- | --- | --- | --- | --- | --- | --- | --- | --- | --- | --- | --- |
| 1 | 22q11.2 Deletion Syndrome | NFC-1 | Chromosomal microdeletion disorder | No | Open-label single-arm trial | Unknown | 12 | Symptomatic improvement | Time to symptom relapse | Pharmaceutical companies | Completed  (2022/3/25) | NCT02895906 |
| 2 | Phelan McDermid Syndrome | Recombinant human Growth hormone | Pathogenic variant in SHANK3 | No | Open-label single-arm trial | Unknown | 10 | Scale-based scores | ABC - Social Withdrawal subscale | Investigators | Published  (2020/6/12) | NCT04003207 |
| 3 | Phelan-McDermid Syndrome | Oxytocin | Pathogenic variant in SHANK3 | No | RCT | Unknown | 40 | Scale-based scores | Change in Aberrant Behavior Checklist | Investigators | Published  (2021/12/6) | NCT02710084 |
| 4 | Adenylosuccinate Lyase Deficiency | Allopurinol | Disorder of purine metabolism | No | Open-label single-arm trial | ＜1/1000000 | 8 | Scale-based scores | composite total score for Vineland II adaptive behaviour Scale | Investigators | Not recruiting  (2021/8/31) | NCT03776656 |
| 5 | Cerebral Adrenoleukodystrophy | Lenti-D | Mutations in the ABCD1 gene and accumulate very long chain fatty acids | Yes | Open-label single-arm trial | 1-9/100000 | 35 | Symptomatic improvement | percentage of Participants who are Alive and Have None of the 6 Major Functional Disabilities | Pharmaceutical companies | Not recruiting  (2022/4/4) | NCT03852498 |
| 6 | Aicardi Goutières Syndrome | Tenofovir and Emtricitabine | Excessive interferon production | Unclear | Randomized crossover trial | 1-5/10000 | 34 | Disease-specific test | Change in interferon activation as measured by interferon response gene | Investigators | Not recruiting  (2022/2/9) | NCT03304717 |
| 7 | Aicardi-Goutières Syndrome | Reverse transcriptase inhibitors | Excessive interferon production | Yes | Open-label single-arm trial | 1-5/10000 | 11 | Disease-specific test | Interferon signature | Investigators | Published  (2019/3/15) | NCT02363452 |
| 8 | Aicardi Goutieres Syndrome | Baricitinib | Excessive interferon production | No | Open-label single-arm trial | 1-5/10000 | 100 | Scale-based scores | Measurement of change with AGS scale at baseline | Investigators | Published  (2022/6/16) | NCT03921554 |
| 9 | Allan-Herndon-Dudley Syndrome | Triac | Mutations in MCT8(a thyroid hormone transporter) | No | Open-label single-arm trial | ＜1/1000000 | 46 | Disease-specific test | Serum T3 concentrations | Investigators | Published  (2019/7/31) | NCT02060474 |
| 10 | Allan-Herndon-Dudley Syndrome | Triac | Mutations in MCT8(a thyroid hormone transporter) | No | Open-label single-arm trial | ＜1/1000000 | 18 | Scale-based scores | Gross Motor Function Measure 88 (GMFM 88) total score | Investigators | Recruiting (2021/6/25) | NCT02396459 |
| 11 | Alpha-Mannosidosis | Velmanase Alfa | Enzyme α-mannosidase defect | Yes | RCT | 1-9/1000000 | 12 | Disease-specific test | Change in concentration of serum oligosaccharides | Pharmaceutical companies | Withdrawn  (2020/12/8) | NCT04031066 |
| 12 | Alternating Hemiplegia | Triheptanoin | Mutations in the ATP1A3 gene | No | Randomized crossover trial | 1/100000 | 10 | Symptomatic improvement | Number of neurologic paroxystic events report in patient diary | Investigators | Published  (2017/10/2) | NCT02408354 |
| 13 | Dermatomyositis | Pirfenidone | Unclear | No | RCT | 1-9/100000 | 57 | Survival rate | changes of 12-month survival from the onset of ILD | Investigators | Not recruiting  (2016/7/1) | NCT02821689 |
| 14 | AADC Deficiency | AAV2-hAADC | Aromatic L-amino acid decarboxylase deficiency | Yes | Open-label single-arm trial | 1/1000000 | 6 | Disease-specific test | CSF neurotransmitter metabolite concentrations | Investigators | Recruiting  (2022/1/31) | NCT02852213 |
| 15 | AADC Deficiency | AAV2-hAADC | Aromatic L-amino acid decarboxylase deficiency | Yes | Open-label single-arm trial | 1/1000000 | 10 | Scale-based scores | PDMS-II score | Investigators | Published  (2017/12/1) | NCT01395641 |
| 16 | AADC Deficiency | AAV2-hAADC | Aromatic L-amino acid decarboxylase deficiency | Yes | Open-label single-arm trial | 1/1000000 | 12 | Disease-specific test | neurotransmitter metabolites | Investigators | Published  (2021/11/8) | NCT02926066 |
| 17 | Ataxia-Telangiectasia | N-Acetyl-L-Leucine | Mutations in the ATM gene | No | Single-arm single-blind trial | 1/100000 | 39 | Symptomatic improvement | Clinical Impression of Change in Severity | Pharmaceutical companies | Recruiting  (2022/8/3) | NCT03759678 |
| 18 | Ataxia Telangiectasia | Vitamin B3 | Mutations in the ATM gene | No | Open-label single-arm trial | 1/100000 | 24 | Scale-based scores | Ataxia, SARA (Scale of the assesment and rating of ataxia) | Investigators | Completed  (2022/7/25) | NCT03962114 |
| 19 | Ataxia Telangiectasia | EDS-EP | Mutations in the ATM gene | Unclear | RCT | 1/100000 | 180 | Scale-based scores | Modified International Cooperative Ataxia Rating Scale | Pharmaceutical companies | Completed  (2021/8/9) | NCT02770807 |
| 20 | Sturge-Weber Syndrome | Cannabidiol | Somatic activating mutation in GNAQ | No | Open-label single-arm trial | 1-9/100000 | 10 | Symptomatic improvement | Number of Seizures Per Month | Investigators | Completed  (2022/3/5) | NCT02332655 |
| 21 | Sturge-Weber Syndrome | Sirolimus | Somatic activating mutation in GNAQ | No | Open-label single-arm trial | 1-9/100000 | 10 | Scale-based scores | Change in Cognitive Function as Assessed by NIH Toolbox Cognitive Battery | Investigators | Completed  (2021/11/1) | NCT03047980 |
| 22 | Centronuclear Myopathies | DYN101 | Mutations in DNM2 or MTM1 gene | Yes | Ranking and selection trial | 1-9/100000 | 18 | Safety | Incidence of drug-related Treatment Emergent Adverse Events | Pharmaceutical companies | Terminated  (2022/7/11) | NCT04033159 |
| 23 | Spinocerebellar Ataxia Type 7 | Riluzole | Unclear | No | Randomized placebo-phase design | 1/1000000 | 34 | Disease-specific test | Visual acuity expressed as log MAR units | Investigators | Unknown | NCT03660917 |
| 24 | Barth syndrome | Elamipretide | Mutations in TAFAZZIN gene | No | Randomized crossover trial | 1/1000000 | 12 | Scale-based scores | Change in distance walked during the 6-minute walk test | Pharmaceutical companies | Completed  (2022/3/9) | NCT03098797 |
| 25 | Duchenne Muscular Dystrophy | L-arginine | Defects in the gene for dystrophin | No | Open-label single-arm trial | 1-9/100000 | 7 | Imaging examinations | MRI/MRS of calf muscle | Investigators | Completed  (2012/7/3) | NCT01388764 |
| 26 | Behcet's Disease | Methylorednisolone | Systemic vasculitis | No | RCT | 1-9/100000 | 34 | Imaging examinations | OCULAR IBDDAM INDEX | Investigators | Unknown | NCT01306955 |
| 27 | Acute Facial Nerve Palsy | Cortisone | Unclear | No | RCT | 1-2/10000 | 500 | Scale-based scores | House-Brackmann scale | Investigators | Recruiting  (2021/8/27) | NCT03781700 |
| 28 | Biotinidase Deficiency | ORL-1B | Biotinidase Deficiency | No | Open-label single-arm trial | 1-9/100000 | 20 | Symptomatic improvement | Improvement in seizure frequency | Investigators | Completed  (2018/1/17) | NCT03269045 |
| 29 | Coats Disease | Bevacizumab | Retinal vascular disease | Unclear | RCT | ＜1/1000000 | 30 | Symptomatic improvement | Proportion of patients with improvement of stage of disease | Investigators | Recruiting  (2021/12/20) | NCT03940690 |
| 30 | Cockayne Syndrome | Prodarsan | Defects in the UV-induced DNA damage repair system | Unclear | Open-label single-arm trial | 1/200000 | 5 | Pharmacokinetics | Pharmacokinetics | Investigators | Completed  (2011/6/23) | NCT01142154 |
| 31 | Congenital Cytomegalovirus | Valganciclovir | Congenital cytomegalovirus infection | Yes | Single-arm single-blind trial | 1-5/10000 | 40 | Symptomatic improvement | Hearing assessment | Investigators | Completed  (2021/6/3) | NCT02005822 |
| 32 | Generalized Lipodystrophy | REGN4461 | Unclear | Unclear | RCT | 1-9/1000000 | 26 | Disease-specific test | Absolute change from baseline hemoglobin A1c | Pharmaceutical companies | Not recruiting  (2022/3/10) | NCT04159415 |
| 33 | Congenital Muscular Dystrophy | Omigapil | Unclear | Unclear | Open-label single-arm trial | 1-9/100000 | 20 | Pharmacokinetics | Pharmacokinetic Profile of Omigapil | Pharmaceutical companies | Completed  (2021/9/24) | NCT01805024 |
| 34 | Childhood Craniopharyngioma | Peginterferon alfa-2b | Tumor | Yes | Open-label single-arm trial | 1-9/100000 | 19 | Imaging examinations | Rate of Disease Stabilization at 1 Year | Investigators | Terminated  (2020/2/24) | NCT01964300 |
| 35 | Adamantinomatous Craniopharyngioma | Tocilizumab | Tumor | No | Open-label single-arm trial | 1-9/100000 | 27 | Safety | Toxicity Profile | Investigators | Recruiting  (2022/5/17) | NCT03970226 |
| 36 | Dermatomyositis | Abatacept | Unclera | Unclear | Open-label single-arm trial | 1-9/100000 | 10 | Scale-based scores | Core Set Measures | Investigators | Completed  (2022/8/29) | NCT02594735 |
| 37 | Dravet Syndrome | GWP42003-P | Mutations in SCN1A gene | No | RCT | 1/28000 | 120 | Symptomatic improvement | Percentage Change From Baseline In Convulsive Seizure Frequency | Pharmaceutical companies | Completed  (2018/8/27) | NCT02091375 |
| 38 | Dravet Syndrome | GWP42003-P | Mutations in SCN1A gene | No | RCT | 1/28000 | 34 | Safety | Number Of Participants who experienced severe adverse Events | Pharmaceutical companies | Published  (2018/3/4) | NCT02091206 |
| 39 | Dravet Syndrome | Fenfluramine HCl | Mutations in SCN1A gene | No | RCT | 1/28000 | 130 | Symptomatic improvement | Change from baseline in frequency of convulsive seizures | Pharmaceutical companies | Published  (2021/10/22) | NCT02826863 |
| 40 | Dravet Syndrome | Fenfluramine HCl | Mutations in SCN1A gene | No | RCT | 1/28000 | 340 | Safety | Long-term safety and tolerability | Pharmaceutical companies | Not recruiting  (2022/9/14) | NCT02823145 |
| 41 | Duchenne Muscular Dystrophy | Vamorolone | Dystrophin deficiency in muscle | No | RCT | 1-9/100000 | 120 | Scale-based scores | Time to Stand Test (TTSTAND) Velocity in Rises/Second | Pharmaceutical companies | Published  (2019/9/24) | NCT03439670 |
| 42 | Duchenne Muscular Dystrophy | PF-06939926 | Dystrophin deficiency in muscle | Yes | RCT | 1-9/100000 | 99 | Scale-based scores | North Star Ambulatory Assessment | Pharmaceutical companies | Recruiting  (2022/9/14) | NCT04281485 |
| 43 | Duchenne Muscular Dystrophy | PF-06939926 | Dystrophin deficiency in muscle | Yes | RCT | 1-9/100000 | 121 | Safety | Number of Participants With Treatment-emergent Adverse Events | Pharmaceutical companies | Terminated  (2020/12/7) | NCT02310763 |
| 44 | Duchenne Muscular Dystrophy | RO7239361 | Dystrophin deficiency in muscle | No | RCT | 1-9/100000 | 166 | Scale-based scores | North Star Ambulatory Assessment (NSAA) Total Score | Pharmaceutical companies | Completed  (2020/12/21) | NCT03039686 |
| 45 | Duchenne Muscular Dystrophy | Vamorolone | Dystrophin deficiency in muscle | No | Randomized placebo-phase design | 1-9/100000 | 120 | Scale-based scores | Time to Stand Test Velocity in Rises/Second Change | Pharmaceutical companies | Published  (2022/8/29) | NCT03439670 |
| 46 | Duchenne Muscular Dystrophy | PF06252616 | Dystrophin deficiency in muscle | No | RCT | 1-9/100000 | 121 | Safety | Number of Participants With Treatment-emergent Adverse Events | Pharmaceutical companies | Terminated  (2020/12/7) | NCT02310763 |
| 47 | Duchenne Muscular Dystrophy | DS-5141b | Dystrophin deficiency in muscle | Yes | Open-label single-arm trial | 1-9/100000 | 7 | Safety | Number of participants with treatment-emergent adverse events | Pharmaceutical companies | Completed  (2020/12/16) | NCT02667483 |
| 48 | Duchenne Muscular Dystrophy | Tamoxifen | Dystrophin deficiency in muscle | No | RCT | 1-9/100000 | 93 | Scale-based scores | MFM D1 subscore | Investigators | Not recruiting  (2022/5/19) | NCT03354039 |
| 49 | Duchenne Muscular Dystrophy | Eteplirsen | Dystrophin deficiency in muscle | Yes | Open-label single-arm trial | 1-9/100000 | 15 | Safety | Number of Participants with Adverse Events | Pharmaceutical companies | Not recruiting  (2022/2/10) | NCT03218995 |
| 50 | Duchenne Muscular Dystrophy | Canakinumab | Dystrophin deficiency in muscle | No | Open-label single-arm trial | 1-9/100000 | 6 | Safety | Clinical adverse events | Investigators | Recruiting  (2022/3/2) | NCT03936894 |
| 51 | Duchenne Muscular Dystrophy | Ataluren | Dystrophin deficiency in muscle | Yes | RCT | 1-9/100000 | 230 | Scale-based scores | Change From Baseline in 6MWD at Week 48 | Pharmaceutical companies | Published  (2017/9/23) | NCT01826487 |
| 52 | Duchenne Muscular Dystrophy | ASP0367 | Dystrophin deficiency in muscle | No | Randomized placebo-phase design | 1-9/100000 | 18 | Safety | Number of participants with Treatment Emergent Adverse Events | Pharmaceutical companies | Recruiting  (2022/9/10) | NCT04184882 |
| 53 | Duchenne Muscular Dystrophy | Metformin and L-citrulline | Dystrophin deficiency in muscle | No | RCT | 1-9/100000 | 40 | Scale-based scores | Mean change of motor function measure (MFM) D1 subscore | Investigators | Published  (2016/8/3) | NCT01995032 |
| 54 | Duchenne Muscular Dystrophy | Viltolarsen | Dystrophin deficiency in muscle | Yes | RCT | 1-9/100000 | 74 | Scale-based scores | Change in Time to Stand | Pharmaceutical companies | Recruiting  (2022/5/25) | NCT04060199 |
| 55 | Duchenne Muscular Dystrophy | SRP-4045/4053 | Dystrophin deficiency in muscle | Yes | RCT | 1-9/100000 | 222 | Scale-based scores | Change in the Total Distance Walked During 6MWT | Pharmaceutical companies | Recruiting  (2022/6/29) | NCT02500381 |
| 56 | Duchenne Muscular Dystrophy | Epigallocatechin-Gallate | Dystrophin deficiency in muscle | No | RCT | 1-9/100000 | 33 | Safety | Safety and tolerability | Investigators | Completed  (2020/7/29) | NCT01183767 |
| 57 | Recurrent Childhood Ependymoma | Everolimus | Tumor | No | Open-label single-arm trial | 1-9/100000 | 11 | Efficacy rate | Objective Response Rate | Investigators | Not recruiting  (2022/2/2) | NCT02155920 |
| 58 | Fabry Disease | Replagal | Lack of alpha-galactosidase activity | Yes | Open-label single-arm trial | 1-5/10000 | 15 | Safety | Number of Serious Adverse Event | Pharmaceutical companies | Published  (2016/5/25) | NCT01363492 |
| 59 | Facioscapulohumeral Muscular Dystrophy | ATYTR1940 | Unclear | Unclear | Open-label single-arm trial | 1-9/100000 | 8 | Safety | Incidences of Treatment-Emergent adverse events and serious adverse events | Pharmaceutical companies | Completed  (2017/5/17) | NCT02603562 |
| 60 | Fabry Disease | Migalastat | Lack of alpha-galactosidase activity | Yes | Open-label single-arm trial | 1-5/10000 | 22 | Safety | Number of Adverse Event | Pharmaceutical companies | Completed  (2021/11/30) | NCT03500094 |
| 61 | Primary Hemophagocytic Lymphohistiocytosis | Emapalumab | Immune dysregulation and hyperinflammation | Yes | Open-label single-arm trial | 1-5/100000 | 34 | Disease-specific test | Achievement of either Complete or Partial Response or HLH Improvement | Pharmaceutical companies | Published  (2020/5/7) | NCT03312751 |
| 62 | Fragile X Syndrome | Ganaxolone | Trinucleotide repeat disorder | No | RCT | 1-5/10000 | 60 | Scale-based scores | Clinician's Global Impression-Improvement | Pharmaceutical companies | Published  (2017/8/2) | NCT01725152 |
| 63 | Fragile X Syndrome | Minocycline hydrochloride | Trinucleotide repeat disorder | No | Randomized crossover trial | 1-5/10000 | 66 | Scale-based scores | Clinical Global Impression Scale | Investigators | Completed  (2017/5/30) | NCT01053156 |
| 64 | Fragile X Syndrome | Mavoglurant | Trinucleotide repeat disorder | No | RCT | 1-5/10000 | 119 | Safety | Incidence and Severity of Adverse Events | Pharmaceutical companies | Terminated  (2016/3/24) | NCT01253629 |
| 65 | Fragile X Syndrome | RO4917523 | Trinucleotide repeat disorder | No | RCT | 1-5/10000 | 47 | Safety | Incidence of adverse events | Pharmaceutical companies | Completed  (2016/8/11) | NCT01750957 |
| 66 | Fragile X Syndrome | STX209 | Trinucleotide repeat disorder | No | RCT | 1-5/10000 | 172 | Scale-based scores | Aberrant Behavior Checklist-Lethargy Social Withdrawal subscale | Pharmaceutical companies | Completed  (2013/7/31) | NCT01325220 |
| 67 | Fragile X Syndrome | Acamprosate | Trinucleotide repeat disorder | No | Open-label single-arm trial | 1-5/10000 | 14 | Scale-based scores | Clinical Global Impression- Severity Scale | Pharmaceutical companies | Completed  (2019/7/30) | NCT01300923 |
| 68 | Fragile X Syndrome | ZYN002 - CBD | Trinucleotide repeat disorder | No | RCT | 1-5/10000 | 212 | Scale-based scores | Aberrant Behavior Checklist-Community Fragile X Factor Structure Social Avoidance Subscale | Pharmaceutical companies | Completed  (2022/7/6) | NCT03614663 |
| 69 | Fragile X Syndrome | Metformin | Trinucleotide repeat disorder | No | Open-label single-arm trial | 1-5/10000 | 15 | Scale-based scores | Change in the total score of the FX-normed Aberrant Behavior Checklist-Community | Investigators | Completed  (2021/2/4) | NCT03722290 |
| 70 | Fragile X Syndrome | NNZ-2566 | Trinucleotide repeat disorder | No | RCT | 1-5/10000 | 72 | Safety | Adverse events | Pharmaceutical companies | Published  (2020/5/23) | NCT01894958 |
| 71 | Friedreich ataxia | IFN-γ | Mutations in FXN gene | No | Open-label single-arm trial | 1-9/100000 | 12 | Disease-specific test | Change in Whole Blood Frataxin Levels | Investigators | Completed  (2021/4/13) | NCT01965327 |
| 72 | Friedreich ataxia | IFN-γ | Mutations in FXN gene | No | RCT | 1-9/100000 | 90 | Scale-based scores | Change in the Friedreich's Ataxia Rating Scale -mNeuro Score | Investigators | Completed  (2018/5/18) | NCT02593773 |
| 73 | Friedreich ataxia | Etravirine | Mutations in FXN gene | No | RCT | 1-9/100000 | 30 | Safety | Adverse event number and severity | Investigators | Not recruiting  (2022/3/31) | NCT04273165 |
| 74 | Friedreich ataxia | RT-001 | Mutations in FXN gene | No | RCT | 1-9/100000 | 60 | Disease-specific test | Change in Maximum Consumption of Oxygen | Pharmaceutical companies | Completed  (2022/5/27) | NCT04102501 |
| 75 | Friedreich ataxia | MIN-102 | Mutations in FXN gene | No | RCT | 1-9/100000 | 36 | Disease-specific test | Change in spinal cord area cervical segment C2-C3 | Pharmaceutical companies | Completed  (2020/9/25) | NCT03917225 |
| 76 | Friedreich ataxia | Epicatechin | Mutations in FXN gene | Yes | Open-label single-arm trial | 1-9/100000 | 10 | Scale-based scores | Change in Friedreich Ataxia Rating Scale | Pharmaceutical companies | Published  (2020/8/31) | NCT02660112 |
| 77 | Friedreich ataxia | Methylprednisolone | Mutations in FXN gene | No | Open-label single-arm trial | 1-9/100000 | 11 | Scale-based scores | Change in the Timed 25 Foot Walk Score | Investigators | Published  (2021/3/25) | NCT02424435 |
| 78 | Friedreich ataxia | EPO | Mutations in FXN gene | No | RCT | 1-9/100000 | 56 | Scale-based scores | Peak oxygen uptake at the cardiopulmonary exercise test | Investigators | Published  (2015/8/11) | NCT01493973 |
| 79 | Gaucher Disease | Eliglustat | Deficiency of the lysosomal enzyme | Yes | Ranking and selection trial | 1-9/100000 | 60 | Safety | Adverse Events | Pharmaceutical companies | Not recruiting  (2022/8/15) | NCT03485677 |
| 80 | Gaucher Disease | Arimoclomol | Deficiency of the lysosomal enzyme | No | RCT | 1-9/100000 | 39 | Disease-specific test | The percentage change in serum chitotriosidase levels | Pharmaceutical companies | Not recruiting  (2021/11/18) | NCT03746587 |
| 81 | Gaucher Disease | ISU302 | Deficiency of the lysosomal enzyme | Unclear | Open-label single-arm trial | 1-9/100000 | 8 | Disease-specific test | The Difference in Hemoglobin Concentration | Investigators | Completed  (2017/7/12) | NCT02770625 |
| 82 | Giant Axonal Neuropathy | scAAV9/JeT-GAN | Shortage of functional Gigaxonin | Yes | Open-label single-arm trial | ＜1/1000000 | 30 | Safety | Adverse event | Investigators | Recruiting  (2022/9/16) | NCT02362438 |
| 83 | GLUT1 Deficiency Syndrome | Triheptanoin | Glucose transporter type-1 deficiency | Yes | Open-label single-arm trial | ＜1/1000000 | 50 | Symptomatic improvement | Reported Change in Seizures Frequency | Pharmaceutical companies | Completed  (2021/1/28) | NCT02036853 |
| 84 | GLUT1 Deficiency Syndrome | UX007 | Glucose transporter type-1 deficiency | No | RCT | ＜1/1000000 | 36 | Symptomatic improvement | Percent reduction in Frequency of Total Seizures | Pharmaceutical companies | Completed  (2020/6/19) | NCT01993186 |
| 85 | GLUT1 Deficiency Syndrome | UX007 | Glucose transporter type-1 deficiency | No | Randomized crossover trial | ＜1/1000000 | 44 | Symptomatic improvement | Maintenance phase movement disorder frequency | Pharmaceutical companies | Terminated  (2020/6/16) | NCT02960217 |
| 86 | GM1 gangliosidosis | LYS-GM101 | Lysosomal storage disorder | Yes | Open-label single-arm trial | ＜1/1000000 | 18 | Symptomatic improvement | Physical examination by body system | Pharmaceutical companies | Not recruiting  (2022/9/2) | NCT04273269 |
| 87 | Spastic paraplegia type 5 | PCSK5 inhibitor | Mutations in the gene CYP7B1 | Yes | Open-label single-arm trial | ＜1/1000000 | 30 | Disease-specific test | The change of 27-hydroxycholesterol | Investigators | Recruiting  (2021/11/23) | NCT04101643 |
| 88 | Hurler syndrome | RGX-111 | Deficiency of α-L-iduronidase | Yes | Open-label single-arm trial | 1-9/1000000 | 5 | Safety | Number of participants with treatment-related adverse events | Pharmaceutical companies | Recruiting  (2022/1/4) | NCT03580083 |
| 89 | Mucopolysaccharidosis I | AGT-181 | Deficiency of α-L-iduronidase | Yes | Open-label single-arm trial | 1-9/1000000 | 21 | Safety | Number of patients with adverse events | Pharmaceutical companies | Published  (2018/7/5) | NCT03053089 |
| 90 | Infantile neuroaxonal dystrophy | RT001 | Mutations in PLA2G6 gene | No | Open-label single-arm trial | ＜1/1000000 | 19 | Scale-based scores | Modified Ashworth Spasticity Scale | Pharmaceutical companies | Not recruiting  (2021/10/14) | NCT03570931 |
| 91 | Infantile neuroaxonal dystrophy | Desipramine | Mutations in PLA2G6 gene | No | Open-label single-arm trial | ＜1/1000000 | 4 | Scale-based scores | Change in Gross Motor Function as Measured by Gross Motor Function Measure | Investigators | Terminated  (2020/10/14) | NCT03726996 |
| 92 | Lennox-Gastaut Syndrome | Rufinamide | Unclear | Unclear | RCT | 1-5/10000 | 37 | Scale-based scores | Child Behavior Checklist Total Problem T-scores | Pharmaceutical companies | Published  (2018/9/1) | NCT01405053 |
| 93 | Lennox-Gastaut Syndrome | GWP42003-P | Unclear | Unclear | Open-label single-arm trial | 1-5/10000 | 116 | Scale-based scores | Change in processing speed on the National Institutes of Health Toolbox Cognition Battery | Pharmaceutical companies | Withdrawn  (2022/9/1) | NCT04133480 |
| 94 | Lennox-Gastaut Syndrome | ZX008 | Unclear | Unclear | RCT | 1-5/10000 | 225 | Symptomatic improvement | Change in frequency of seizures that result in drops in subjects receiving ZX008 | Pharmaceutical companies | Not recruiting  (2022/7/30) | NCT03355209 |
| 95 | Lennox-Gastaut Syndrome | Rufinamide | Unclear | Unclear | RCT | 1-5/10000 | 66 | Symptomatic improvement | Percent Change in Tonic-Atonic Seizure Frequency | Pharmaceutical companies | Published  (2020/11/8) | NCT01146951 |
| 96 | Lennox-Gastaut Syndrome | GWP42003 | Unclear | Unclear | RCT | 1-5/10000 | 225 | Symptomatic improvement | Percentage Change In Drop Seizure Frequency | Pharmaceutical companies | Published  (2018/5/17) | NCT02224560 |
| 97 | Lennox-Gastaut Syndrome | Carisbamate | Unclear | Unclear | Open-label single-arm trial | 1-5/10000 | 30 | Symptomatic improvement | Seizure Frequency | Pharmaceutical companies | Not recruiting  (2022/5/16) | NCT04062981 |
| 98 | Lennox-Gastaut Syndrome | Perampanel | Unclear | Unclear | RCT | 1-5/10000 | 142 | Symptomatic improvement | Median Percent Change in Drop Seizure Frequency | Pharmaceutical companies | Terminated  (2022/3/9) | NCT02834793 |
| 99 | Lesch-Nyhan Disease | Ecopipam | Unclear | No | Randomized crossover trial | 1-9/1000000 | 24 | Scale-based scores | Behavior Problems Inventory Self-InjuriousBehavior Subscale | Pharmaceutical companies | Terminated  (2015/10/8) | NCT01751802 |
| 100 | Limb-Girdle Muscular Dystrophy | SRP-9003 | Unclear | Unclear | Open-label single-arm trial |  | 6 | Safety | Number of Treatment-Emergent Adverse Events | Pharmaceutical companies | Not recruiting  (2021/6/8) | NCT03652259 |
| 101 | MELAS Syndrome | L-Arginine | Impaired mitochondrial translation and protein synthesis | No | Open-label single-arm trial | 1-9/1000000 | 9 | Imaging examinations | Muscle function investigation via 31P-Magnetic resonance spectroscopy | Investigators | Published  (2020/9/3) | NCT01603446 |
| 102 | Molybdenum Cofactor Deficiency | ORGN001 | Molybdenum cofactor deficiency | No | Open-label single-arm trial | ＜1/1000000 | 5 | Symptomatic improvement | Overall survival | Pharmaceutical companies | Recruiting  (2022/9/1) | NCT02629393 |
| 103 | Mucopolysaccharidosis Type IIIB | AX250 | Lysosomal storage disease | Unclear | Open-label single-arm trial | 1-9/1000000 | 23 | Safety | Number of participants with abnormal clinical laboratory values | Pharmaceutical companies | Completed  (2020/8/6) | NCT02754076 |
| 104 | Mucopolysaccharidosis Type IIIA | SOBI003 | Lysosomal storage disease | Yes | Open-label single-arm trial | 1-9/1000000 | 6 | Safety | Safety as Measured by Adverse Events Frequencies | Pharmaceutical companies | Published  (2022/6/8) | NCT03423186 |
| 105 | Mucopolysaccharidosis Type IIIB | SBC-103 | Lysosomal storage disease | Yes | Open-label single-arm trial | 1-9/1000000 | 11 | Safety | Number Of Participants Who Experienced Severe Treatment-emergent Adverse Events | Pharmaceutical companies | Published  (2018/12/3) | NCT02324049 |
| 106 | Mucopolysaccharidosis Type IIIB | rAAV9.CMV.hNAGLU | Lysosomal storage disease | Yes | Open-label single-arm trial | 1-9/1000000 | 15 | Safety | The incidence, type and severity of treatment-related adverse events and serious adverse events | Pharmaceutical companies | Terminated  (2022/5/5) | NCT03315182 |
| 107 | Myasthenia Gravis | Eculizumab | Autoimmune disorder | No | Open-label single-arm trial | 1-9/1000000 | 12 | Scale-based scores | Change in the QMG Total Score | Pharmaceutical companies | Not recruiting  (2020/8/25) | NCT03759366 |
| 108 | Congenital Myotonic Dystrophy | Tideglusib | Unclear | Unclear | RCT | 1-9/1000000 | 56 | Scale-based scores | Clinician-Completed Congenital DM1 Rating Scale | Pharmaceutical companies | Recruiting  (2022/8/15) | NCT03692312 |
| 109 | Narcolepsy | Armodafinil | Unclear | Unclear | RCT | 1-5/10000 | 40 | Pharmacokinetics | Pharmacokinetics | Pharmaceutical companies | Completed  (2021/11/9) | NCT01624480 |
| 110 | Narcolepsy | Pitolisant | Unclear | Unclear | RCT | 1-5/10000 | 96 | Scale-based scores | Changes in EDS measured by the Ullanlinna Narcolepsy Scale Score | Pharmaceutical companies | Not recruiting  (2022/1/14) | NCT02611687 |
| 111 | Neurofibromatosis Type 1 | Lamotrigine | Mutations in NF1 gene | No | RCT | 1-5/10000 | 41 | Scale-based scores | Performance intelligence quotient | Investigators | Terminated  (2020/4/14) | NCT02256124 |
| 112 | Neurofibromatosis Type 1 | Trametinib | Mutations in NF1 gene | No | Open-label single-arm trial | 1-5/10000 | 15 | Imaging examinations | Remission of tumor volume ≥20% | Pharmaceutical companies | Not recruiting  (2022/4/28) | NCT03741101 |
| 113 | Neurofibromatosis Type 2 | Everolimus | Mutations in NF2 gene | Yes | Open-label single-arm trial | 1-9/100000 | 10 | Imaging examinations | Radiographic Response | Pharmaceutical companies | Completed  (2017/7/18) | NCT01419639 |
| 114 | NMOSD | Eculizumab | Unclear | Unclear | Open-label single-arm trial | 1-9/100000 | 15 | Symptomatic improvement | Change In The Annualized Relapse Rate | Pharmaceutical companies | Recruiting  (2022/4/13) | NCT04155424 |
| 115 | NMOSD | Satralizumab | Unclear | Unclear | RCT | 1-9/100000 | 83 | Symptomatic improvement | Time to First Protocol-Defined Relapse | Pharmaceutical companies | Published  (2022/7/5) | NCT02028884 |
| 116 | CLN3 Batten Disease | AT-GTX | Lysosomal storage disease | Yes | Open-label single-arm trial | ＜1/1000000 | 7 | Safety | The development of dose-limiting toxicity | Pharmaceutical companies | Not recruiting  (2022/7/20) | NCT03770572 |
| 117 | Late Infantile Neuronal Ceroid Lipofuscinosis | AAVrh.10CUhCLN2 vector | Lysosomal storage disease | Yes | Open-label single-arm trial | ＜1/1000000 | 25 | Scale-based scores | Change in Weill-Cornell LINCL scale | Investigators | Completed  (2021/2/2) | NCT01161576 |
| 118 | CLN2 Disease | BMN 190 | Lysosomal storage disease | Yes | Open-label single-arm trial | ＜1/1000000 | 23 | Scale-based scores | Probability of Unreversed 2-point Decline in Motor-language Score | Pharmaceutical companies | Completed  (2022/8/24) | NCT02485899 |
| 119 | Niemann-Pick Disease, Type C | VTS-270 | Lysosomal storage disease | No | Open-label single-arm trial | 1-9/100000 | 12 | Disease-specific test | Reduce plasma levels of glycine-conjugated trihydroxycholanic acid | Investigators | Not recruiting  (2022/4/12) | NCT03471143 |
| 120 | Niemann-Pick Disease, Type C | Arimoclomol | Lysosomal storage disease | No | RCT | 1-9/100000 | 50 | Scale-based scores | Change in NPC disease severity score | Pharmaceutical companies | Not recruiting  (2021/4/19) | NCT02612129 |
| 121 | Niemann-Pick Disease, Type C | Lithium Carbonate | Lysosomal storage disease | No | Open-label single-arm trial | 1-9/100000 | 18 | Scale-based scores | NPC clinical severity score | Investigators | Not recruiting  (2019/9/24) | NCT03201627 |
| 122 | Niemann-Pick Disease, Type C | N-Acetyl-L-Leucine | Lysosomal storage disease | No | Open-label single-arm trial | 1-9/100000 | 34 | Scale-based scores | Clinical Impression of Change in Severity | Pharmaceutical companies | Not recruiting  (2022/8/3) | NCT03759639 |
| 123 | Niemann-Pick Disease, Type C | Miglustat | Lysosomal storage disease | No | Open-label single-arm trial | 1-9/100000 | 18 | Scale-based scores | Observed change in HSEM | Pharmaceutical companies | Completed  (2022/5/2) | NCT03910621 |
| 124 | Noonan Syndrome | HMG-COA Reductase Inhibitor | Mutations in the PTPN11 gene | No | RCT | 1-5/10000 | 62 | Disease-specific test | Change in Insulin-like Growth Factor-1 | Investigators | Recruiting  (2020/11/9) | NCT02713945 |
| 125 | Noonan Syndrome | Somatropin | Mutations in the PTPN11 gene | No | RCT | 1-5/10000 | 51 | Symptomatic improvement | Change in Height SDS | Pharmaceutical companies | Published  (2020/5/9) | NCT01927861 |
| 126 | Osteoporosis-pseudoglioma Syndrome | Lithium | Mutations in the LRP5 gene |  | Open-label single-arm trial | ＜1/1000000 | 26 | Imaging examinations | pQCT of Lower Leg | Investigators | Completed  (2019/11/4) | NCT01108068 |
| 127 | Pantothenate kinase-associated neurodegeneration | CoA-Z | Mutations in the PANK2 gene | Unclear | Randomized crossover trial | 2-3/1000000 | 51 | Safety | Number of participants experiencing adverse events | Investigators | Not recruiting  (2022/2/9) | NCT04182763 |
| 128 | Pantothenate kinase-associated neurodegeneration | Deferiprone | Mutations in the PANK2 gene | Unclear | Randomized placebo-phase design | 2-3/1000000 | 89 | Scale-based scores | Change in Score on Barry-Albright Dystonia Scale | Pharmaceutical companies | Published  (2019/7/18) | NCT01741532 |
| 129 | Pantothenate kinase-associated neurodegeneration | RE-024 | Mutations in the PANK2 gene | Yes | RCT | 2-3/1000000 | 84 | Scale-based scores | Change In The Pantothenate Kinase-Associated Neurodegeneration-Activities of Daily Living Total Score | Pharmaceutical companies | Terminated  (2021/1/26) | NCT03041116 |
| 130 | Rett Syndrome | NNZ-2566 | Mutations in the MECP2 gene | No | RCT | 1-9/100000 | 82 | Safety | Adverse events | Pharmaceutical companies | Completed  (2020/8/14) | NCT02715115 |
| 131 | Rett Syndrome | IGF-1 | Mutations in the MECP2 gene | No | Randomized crossover trial | 1-9/100000 | 30 | Scale-based scores | Rett Syndrome Behavior Questionnaire | Investigators | Published  (2014/3/12) | NCT01777542 |
| 132 | Rett Syndrome | Ketamin | Mutations in the MECP2 gene | No | Randomized crossover trial | 1-9/100000 | 48 | Safety | Dose-Limiting Adverse Events | Investigators | Completed  (2022/4/22) | NCT03633058 |
| 133 | Rett Syndrome | Glatiramer Acetate | Mutations in the MECP2 gene | No | Open-label single-arm trial | 1-9/100000 | 10 | Imaging examinations | Improvement of epileptiform activity | Pharmaceutical companies | Recruiting  (2014/2/4) | NCT02023424 |
| 134 | Rett Syndrome | Dextromethorphan | Mutations in the MECP2 gene | No | RCT | 1-9/100000 | 57 | Scale-based scores | Change in Mullen; Visual Reception Sub-scale Score | Investigators | Completed  (2018/12/4) | NCT01520363 |
| 135 | Rett Syndrome | RETT-T | Mutations in the MECP2 gene | No | Randomized crossover trial | 1-9/100000 | 35 | Scale-based scores | Rett Syndrome Natural History Motor Behavior Assessment | Investigators | Not recruiting  (2019/8/13) | NCT04041713 |
| 136 | Rett Syndrome | EPI-743 | Mutations in the MECP2 gene | No | RCT | 1-9/100000 | 24 | Scale-based scores | Rett Syndrome Clinical Severity Sore | Pharmaceutical companies | Completed  (2018/7/26) | NCT01822249 |
| 137 | Rett Syndrome | ANAVEX2-73 | Mutations in the MECP2 gene | Yes | RCT | 1-9/100000 | 69 | Scale-based scores | Rett Syndrome Behaviour Questionnaire | Pharmaceutical companies | Recruiting  (2022/9/7) | NCT04304482 |
| 138 | Rett Syndrome | Trofinetide | Mutations in the MECP2 gene | No | RCT | 1-9/100000 | 184 | Scale-based scores | Rett Syndrome Behaviour Questionnaire | Pharmaceutical companies | Published  (2022/2/8) | NCT04181723 |
| 139 | Rett Syndrome | GWP42003-P | Mutations in the MECP2 gene | No | RCT | 1-9/100000 | 252 | Scale-based scores | Rett Syndrome Behaviour Questionnaire | Pharmaceutical companies | Terminated  (2022/9/2) | NCT03848832 |
| 140 | Rett Syndrome | Sarizotan | Mutations in the MECP2 gene | No | RCT | 1-9/100000 | 129 | Symptomatic improvement | Reduction in Respiratory Abnormality | Pharmaceutical companies | Terminated  (2021/12/21) | NCT02790034 |
| 141 | Sandhoff Disease | GZ402671 | Mutations in the HEXB gene | No | RCT | 1-9/100000 | 62 | Disease-specific test | Change in cerebrospinal fluid (CSF) GM2 biomarker | Pharmaceutical companies | Not recruiting  (2022/7/8) | NCT04221451 |
| 142 | Spinal Muscular Atrophy Type I | Onasemnogene Abeparvovec-xioi | Mutations in the SMN1 gene | Yes | Open-label single-arm trial | 1-9/100000 | 6 | Symptomatic improvement | Number of Participants Who Achieved Sitting Alone for at Least 10 Seconds | Pharmaceutical companies | Completed  (2022/8/16) | NCT03837184 |
| 143 | Spinal Muscular Atrophy Type I | AVXS-101 | Mutations in the SMN1 gene | Yes | Open-label single-arm trial | 1-9/100000 | 15 | Safety | Number of Participants That Experienced Treatment-related Toxicity | Pharmaceutical companies | Published  (2019/3/16) | NCT02122952 |
| 144 | Spinal Muscular Atrophy | Nusinersen | Mutations in the SMN1 gene | Yes | RCT | 1-9/100000 | 126 | Scale-based scores | Hammersmith Functional Motor Scale | Pharmaceutical companies | Completed  (2021/2/17) | NCT02292537 |
| 145 | Spinal Muscular Atrophy | RO7034067 | Mutations in the SMN1 gene | Yes | Open-label single-arm trial | 1-9/100000 | 174 | Safety | Percentage of Participants With Adverse Events and Serious AEs | Pharmaceutical companies | Not recruiting  (2022/8/3) | NCT03032172 |
| 146 | Spinal Muscular Atrophy | Risdiplam | Mutations in the SMN1 gene | Yes | Open-label single-arm trial | 1-9/100000 | 25 | Disease-specific test | Percentage of participants sitting without support | Pharmaceutical companies | Not recruiting  (2022/7/29) | NCT03779334 |
| 147 | Spinal Muscular Atrophy | Olesoxime | Mutations in the SMN1 gene | No | RCT | 1-9/100000 | 165 | Scale-based scores | Motor Function Measure D1+D2 score | Pharmaceutical companies | Published  (2017/4/28) | NCT01302600 |
| 148 | Spinal Muscular Atrophy | SRK-015 | Mutations in the SMN1 gene | No | RCT | 1-9/100000 | 58 | Scale-based scores | Change in the Revised Hammersmith Scale | Pharmaceutical companies | Not recruiting  (2021/6/18) | NCT03921528 |
| 149 | Spinal Muscular Atrophy | CK-2127107 | Mutations in the SMN1 gene | No | RCT | 1-9/100000 | 70 | Disease-specific test | Change in Forced Vital Capacity | Pharmaceutical companies | Completed  (2020/8/31) | NCT02644668 |
| 150 | Spinal Muscular Atrophy | Celecoxib | Mutations in the SMN1 gene | No | Open-label single-arm trial | 1-9/100000 | 12 | Disease-specific test | Increase in the levels of peripheral leukocyte SMN protein | Investigators | Terminated  (2020/10/19) | NCT02876094 |
| 151 | Spinal Muscular Atrophy | Amifampridine Phosphate | Mutations in the SMN1 gene | No | Randomized crossover trial | 1-9/100000 | 12 | Scale-based scores | Hammersmith Functional Motor Scale | Pharmaceutical companies | Published  (2022/6/28) | NCT03781479 |
| 152 | Succinic Semialdehyde Dehydrogenase Deficiency | SGS-742 | Succinic Semialdehyde Dehydrogenase Deficiency | No | Randomized crossover trial | ＜1/1000000 | 19 | Scale-based scores | Change on the Adaptive Behavior Assessment System (ABAS) Test | Investigators | Published  (2021/5/20) | NCT02019667 |
| 153 | Tay-Sachs Disease | Venglustat | Deficiency of the enzyme β-N-acetylhexosaminidase A | No | RCT | ＜1/1000000 | 62 | Disease-specific test | Change in cerebrospinal fluid (CSF) GM2 biomarker | Pharmaceutical companies | Not recruiting  (2022/7/8) | NCT04221451 |
| 154 | Tay-Sachs Disease | IB001 | Deficiency of the enzyme β-N-acetylhexosaminidase A | Yes | Open-label single-arm trial | ＜1/1000000 | 39 | Scale-based scores | Clinical Impression of Change in Severity | Pharmaceutical companies | Not recruiting  (2022/8/3) | NCT03759665 |
| 155 | Tuberous Sclerosis | Vigabatrin | Dysfunction of hamartin or tuberin | No | RCT | 1-5/10000 | 84 | Scale-based scores | Cognitive Assessment Scores and Developmental Impact | Investigators | Not recruiting  (2022/5/6) | NCT02849457 |
| 156 | Tuberous Sclerosis | Everolimus | Dysfunction of hamartin or tuberin | Yes | RCT | 1-5/10000 | 60 | Scale-based scores | Cognitive ability measured by IQ | Investigators | Published  (2019/6/19) | NCT01730209 |
| 157 | Tuberous Sclerosis | Everolimus | Dysfunction of hamartin or tuberin | Yes | RCT | 1-5/10000 | 96 | Symptomatic improvement | Facial Angiofibroma Severity Index | Investigators | Withdrawn  (2021/5/3) | NCT02860494 |
| 158 | Tuberous Sclerosis | Everolimus | Dysfunction of hamartin or tuberin | Yes | RCT | 1-5/10000 | 366 | Symptomatic improvement | Seizure Frequency Response Rate | Pharmaceutical companies | Published  (2018/5/24) | NCT01713946 |
| 159 | Tuberous Sclerosis | GWP42003-P | Dysfunction of hamartin or tuberin | No | RCT | 1-5/10000 | 224 | Symptomatic improvement | Percent Change in the Number of Tuberous Sclerosis Complex (TSC)-Associated Seizures | Pharmaceutical companies | Published  (2021/3/1) | NCT02544763 |
| 160 | Infantile Spasm | Fenfluramine | Unclear | No | Open-label single-arm trial | 1-9/100000 | 10 | Imaging examinations | Electroclinical response | Investigators | Not recruiting  (2020/2/28) | NCT04289467 |
| 161 | Infantile Spasm | Cannabidiol | Unclear | No | RCT | 1-9/100000 | 190 | Symptomatic improvement | Percentage of Participants Considered Complete Responders | Pharmaceutical companies | Terminated  (2021/11/13) | NCT03421496 |
| 162 | Infantile Spasm | JBPOS0101 | Unclear | Unclear | Open-label single-arm trial | 1-9/100000 | 35 | Safety | Incidence and severity of treatment emergent adverse events | Pharmaceutical companies | Terminated  (2022/7/26) | NCT03976076 |
